# Long Period Grating Modified with Quasi-2D Perovskite/PAN Hybrid Nanofibers for Relative Humidity Measurement

**DOI:** 10.3390/nano16020099

**Published:** 2026-01-12

**Authors:** Dingyi Feng, Changjiang Zhang, Syed Irshad Haider, Jing Tian, Jiandong Wu, Fu Liu, Biqiang Jiang

**Affiliations:** 1 MOE Key Laboratory of Material Physics and Chemistry under Extraordinary Conditions, State Key Laboratory of Porous Metal Materials, Shaanxi Basic Discipline (Liquid Physics) Research Center, and School of Physical Science and Technology, Northwestern Polytechnical University, Xi’an 710129, China; nupzcj@mail.nwpu.edu.cn (C.Z.); syed.irshad@mail.nwpu.edu.cn (S.I.H.); tianj@mail.nwpu.edu.cn (J.T.); fu.liu@nwpu.edu.cn (F.L.); bqjiang@nwpu.edu.cn (B.J.); 2 State Key Laboratory of Solidiffcation Processing, Center for Nano Energy Materials, School of Materials Science and Engineering, Northwestern Polytechnical University and Shaanxi Joint Laboratory of Graphene, Xi’an 710129, China; jiandong.wu@mail.nwpu.edu.cn

**Keywords:** optical fiber, long period grating, perovskite materials, nanofibers, humidity sensing

## Abstract

Metal halide perovskites have emerged as promising photoactive materials for highly efficient photodetectors; however, the inherent instability of perovskite materials in oxygen and moisture limits their practical applications. In this study, the highly moisture-sensitive characteristics of the quasi-2D perovskite nanocrystals were used to fabricate a long-period grating (LPG) humidity sensor based on the perovskite/polyacrylonitrile (PAN) hybrid nanofibers film. The pure-bromide quasi-2D perovskite nanocrystals were in situ synthesized and encapsulated in the PAN matrix on the fiber grating via an electrospinning technique. Humidity-induced variation in the complex permittivity of perovskites can alter the evanescent field of the co-propagating cladding modes, resulting in changes in both resonant amplitude and wavelength in the transmission spectrum of the LPG. These effects yielded an intensity sensitivity of ~0.21 dB/%RH and a wavelength sensitivity of ~18.2 pm/%RH, respectively, in the relative humidity range of 50–80%RH. The proposed LPG sensor demonstrated a good performance, indicating its potential application in the humidity-sensing field.

## 1. Introduction

Humidity is a critical parameter as it directly affects human health, personal comfort, industrial production efficiency, and the performance of technological devices [1]. Traditional electronic humidity sensors (i.e., capacitive and resistive ones) show several advantages such as high accuracy, low cost, fast response and good linearity; however, they have drawbacks of corrosion, susceptibility in harsh environment and electromagnetic interference. In the past decades, fiber-optic sensors have emerged as a promising technique for a wide range of applications owing to the inherent advantages of small size, light weight, immunity to electromagnetic interference, biocompatibility and survivability in hazardous/corrosive environment. Various fiber humidity sensors have been developed by using Fabry–Perot interferometer [2,3], Mach–Zehnder interferometer [4], tapered fiber [5], photonic crystal fibers [6], and fiber grating [7,8,9].

Among these sensors, fiber gratings are well established as point- and quasi-distributed humidity sensing elements that do not require complex fabrication processes such as fiber etching or misalignment [10]. Gratings are periodic structures formed in the fiber core, enabling the interaction of incident light with surrounding medium. Fiber-optic grating technology has evolved to include several grating types, including fiber Bragg gratings (FBGs), long-period fiber gratings (LPGs), and tilted fiber gratings (TFGs). In addition to these fiber-based configurations, long-period gratings based on integrated waveguide platforms have also been demonstrated for sensing applications in recent years. Nevertheless, fiber gratings continue to attract widespread attention in humidity sensing due to their structural flexibility, ease of integration with functional materials, capability for distributed or quasi-distributed sensing, and good compatibility with existing optical fiber systems [11,12]. Since FBGs are inherently insensitive to humid environments, various hydrophilic polymeric coatings can be integrated with gratings, where the water-absorbing nature of the polymers induces physical swelling of the fiber structure [13]. LPGs and TFBGs exhibit enhanced sensing performance when integrated with varies moisture-sensitive materials such as metal oxide and graphene oxide, because the variation in water vapor concentration can alter the complex permittivity of the coated films, leading to changes in the effective refractive index (RI) of higher-order cladding modes of the grating that interact with the medium. Researchers have made extensive efforts in identifying various sensing materials for optical fiber humidity sensors based on fiber gratings in recent years. For instance, Tsai et al. proposed a graphene oxide (GO)-coated S-type long period fiber grating (LPG) in 2020 [14]. The abundance of oxygen-containing functional groups on the surface of GO contributes to humidity response since it can permeate and absorb more water molecules. After analyzing the resonance wavelength shift and the transmission loss of the resonance dip for different grating diameters, they obtained a highest sensitivity at 0.18 dB/%RH. Wang et al. proposed a TFBG sensor coated with GO/multi-walled carbon nanotubes (CNTs) hybrid nanomaterial in 2021 [15]. This three-dimensional structural nanomaterial can substantially enhance the detection sensitivity by up to 0.377 dB/%RH. Peng et al. reported a humidity sensor incorporating a FBG coated with a porous anodic alumina film via anodic oxidation in 2023 [16]. This sensor showed a humidity sensitivity of 18.5 pm/%RH in wavelength. Jing et al. exposed an agarose-coated TFG sensor, and also monitored the resonance wavelength to test its humidity-sensing capabilities in 2024 [9]. It demonstrated that the sensor exhibited a rapid response time and high response sensitivity of 18.5 pm/%RH. Li et al. designed a humidity sensor based on phase-shifted FBG with polymethylmethacrylate (PMMA) microsphere Fabry–Perot (FP) cavity in 2025 [17]. PMMA and other polymers, such as polyvinyl alcohol (PVA) and polyimide (PI), exhibit excellent film-forming capabilities and can be combined with various nanoparticles—including metal oxide semiconductors, graphene quantum dots (GQDs), and carbon nanotubes (CNTs)—to form hybrid nanomaterials, which hold significant potential for high-sensitivity humidity sensing applications [18,19,20].

In this study, we prepared an LPG sensor based on quasi-2D perovskite/ polyacrylonitrile (PAN) hybrid nanofibers for humidity sensing. Perovskite served as an excellent humidity-sensitive material, as the water molecules infiltrated the perovskite lattice and interacted with perovskite ions via hydrogen bonding, and a substantial number of surface and bulk defects could be generated, leading to changes in RI of materials [21,22,23]. Although perovskites are generally not stable in air, by mixing the perovskite precursor in the polyacrylonitrile (PAN) solution and high-voltage electrospinning processing, the pure-bromide quasi-2D perovskite nanocrystals were in situ synthesized and encapsulated in the PAN matrix, which avoided the tedious synthesis, purification, and protection of perovskites. Via optimizing the concentration of the perovskite in PAN electrospinning matrix, the hybrid nanofibers film provided the abundant and unimpeded water molecule transport channels. Considering the strong adhesion of PAN, the hybrid nanofibers were subsequently coated onto the surface of LPG to functionalize the grating for RH measurement. Compared with FBG and TFBG [24,25,26], LPG exhibits higher sensitivity to external RI changes, as the evanescent field of the coupled cladding modes is directly exposed to the external environment [27,28]. The experimental results showed that humidity-induced complex permittivity changes on the perovskite/PAN hybrid nanofibers film can significantly modify the evanescent field of the guided cladding modes, and directly result in the change both in wavelength and amplitude in resonant peaks of the grating. This study may provide a simpler and universal platform for large-scale construction of stable luminescent materials and devices. Moreover, it provides an effective solution for humidity monitoring.

## 2. Materials and Characterization

**Figure 1:**
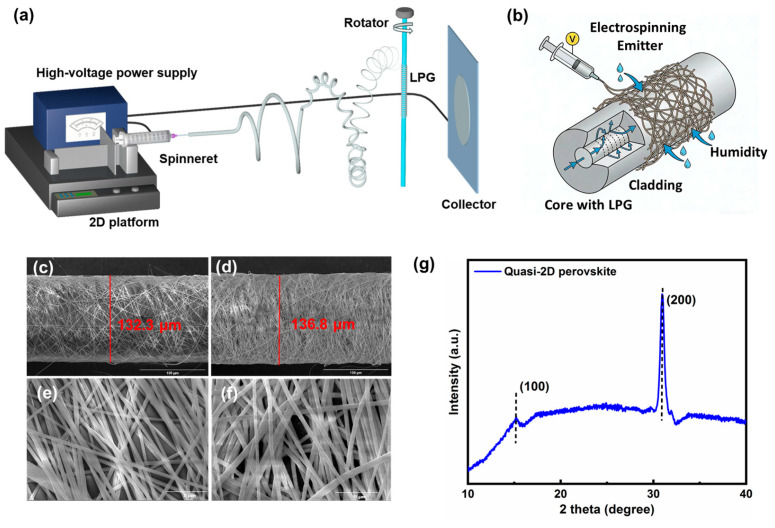
Schematic diagrams of electrospinning process for preparing perovskite/PAN hybrid nanofibers (**a**) and the LPG sensing structure (**b**); SEM images of the film wrapped on LPG prepared by electrospinning method: perovskite/PAN nanofibers with electrospun times of (**c**,**e**) 50 s and (**d**,**f**) 3 min, respectively; XRD patterns of quasi-2D perovskite materials (**g**).

The quasi-2D perovskite/PAN hybrid nanofibers around fiber grating were fabricated via an electrospinning technique, as illustrated in Figure 1a,b. Different from traditional methods for preparing nanofibers—such as template synthesis, self-assembly, melt-blowing, and phase separation—electrospinning is a much simpler and cost-effective technique for producing nanofibers of organic or inorganic materials. Herein, the perovskite precursor solution is a mixture of A (PEA_2_PbBr_4_/DMSO) and solution B (CsPbBr_3_/DMSO) in a volume ratio of 2:1. Solution A and solution B were prepared by dissolving CsBr, PEABr, and PbBr_2_ in DMSO, maintaining the molar concentration of PbBr_2_ at 0.1 M, and stirring continuously for 3 h at room temperature. Subsequently, 0.5 M β-alanine was added to the mixed solution, followed by continuous stirring for 2 h at 80 °C. Then PAN was dissolved in DMSO with a concentration of 8 wt%. Finally, the perovskite precursor solution and PAN solution were mixed, stirring continuously for 3 h at room temperature. The purity of the materials for configuring the perovskite precursors are all 99.999%, and the materials CsBr, PEABr, PbBr_2,_ and DMSO used in the precursor solution are all from Sigma (St. Louis, MO, USA).

During the electrospinning process, the hydrolysis, condensation, and gelation of the polymer significantly influence the morphological and microstructural evolution of the nanofibers. As shown in the schematic diagram of Figure 1a, the prepared precursor solution was extruded by a syringe, and installed on the syringe automatic propulsion platform with a speed of 0.3 mL/h. The needle of the syringe was faced toward the collector, and a high voltage of 17 kV was applied between the needle (positive electrode) and the collector (negative electrode) to convert the solution into nanofibers. An optical fiber was used as the substrate, and fixed in front of the collector and driven by a rotating motor with a speed of ~230 r/min. Under these conditions, high-quality and reproducible perovskite/PAN nanofibers were successfully wrapped on the surface of the LPG.

The schematic diagram of LPG modified with hybrid nanofibers film is shown in Figure 1b. An LPG with grating pitch of 550 μm and a length of 10 mm was employed as the humidity sensing element. The wavelength of the grating is 1550 nm, and loss-band depth is −25 dB. The essence of LPG is that the light generated from the core mode is coupled to the cladding mode whose wavelength satisfies the phase matching condition. The resonant wavelength that describes the coupling between core mode and *m*th evanescent cladding mode is expressed as follows [28]:

(1) $$\lambda_{\mathrm{res}}=(n_{eff,co}-n_{eff,cl}^{m})\Lambda$$

where Λ is the grating pitch of the LPG. $n_{eff,co}$ and $n_{eff,cl}^{m}$ represent the effective RI of the core fundamental mode and the *m*th-order cladding modes, respectively. The working principle of the designed sensor is based on the analysis of light propagating in the cladding modes, affected by the wrapped hybrid nanofibers. As water molecules penetrate the quasi-2D perovskite/PAN hybrid nanofibers, the effective RI difference in the core and cladding and the pitch of the grating will both change, correspondingly. On the other hand, the guided modes in the cladding will partially act as leaky cladding modes, and the change in the intensity of resonance in the LPG is governed by the equation as follows:

(2) $$T_{m}=1-\sin^{2}(K_{m}L)$$

where *T_m_* is the amplitude of the loss band corresponding to the coupling of the fundamental core mode to the *m*th cladding mode. *K_m_* and *L* are the coupling coefficient and length of the LPG. As explained above, the RI change induced by changes in the surrounding environment affects the wavelength and evanescent filed of the LPG loss band.

The scanning electron microscope (SEM) images of the prepared perovskite/PAN hybrid nanofibers on fiber grating surface with different thickness are presented in Figure 1c,d. It can be seen that the hybrid nanofibers are densely coated on the surface of the optical fiber. The surface roughness of the film can be directly attributed to its porous microstructure. The bare optical fiber has a diameter of 125.5 µm, and the measured thicknesses of the hybrid nanofibers film on the fiber surface are 6.8 µm (Figure 1c) under electrospun time of 50 s, and 11.3 µm under electrospun time of 3 min (Figure 1d), respectively. Enlarged images of Figure 1c,d shown in Figure 1e,f revealed the porous reticular film morphology on the surface of the grating, and it can also be observed that perovskite crystals are effectively formed and embedded within the PAN matrix. To further confirm the presence of quasi-2D perovskite, the XRD phase analysis of the hybrid nanofibers was recorded and shown in Figure 1g. It can be seen that the diffraction peaks appeared at 16° and 31°, corresponding to the (1 0 0) plane and (2 0 0) plane of the material, respectively. They both match well with reference XRD data for cubic CsPbBr_3_ (PDF-00-054-0752) and the reference [29]. It convincingly demonstrates the formation of perovskite nanocrystals within the PAN nanofibers [30].

## 3. Results and Discussion

**Figure 2:**
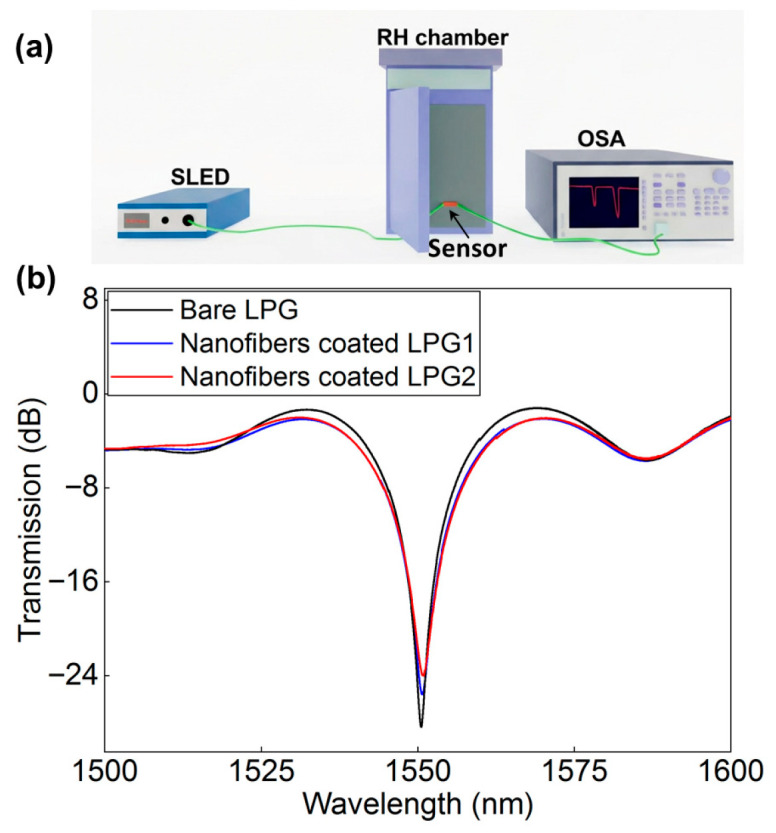
Schematic diagram of the relative humidity experimental system (**a**) and the transmission spectra of LPG before and after being encapsulated by quasi-two-dimensional perovskite/PAN hybrid nanofibers at an environmental relative humidity of 50% (**b**).

The schematic diagram of the relative humidity experimental system are shown in Figure 2a. A constant temperature and humidity chamber was used as the relative humidity control unit, which provides humidity levels from 50% to 80% under constant temperature conditions of 35 °C. A broadband source (SLED-1550) was utilized as the incident light, and the transmission spectra of the sensor were recorded by an optical spectrometer (OSA) with a resolution of 0.01 nm.

During the RH experiment, two kinds of grating samples were fabricated with perovskite precursor and PAN solution at volume ratios of 1:10 (LPG1) and 2:10 (LPG2), respectively. Figure 2b depicts the transmitted spectra of the LPGs before and after deposition of the perovskite/PAN hybrid nanofibers. It can be observed that the transmission spectral intensity of the grating was clearly reduced after deposition of the hybrid nanofibers on its surface. Meanwhile, by comparing the transmission spectra of LPG1 and LPG2, it is evident that the perovskite ratio influences the loss band of the grating. As the doping ratio increased from 1:10 to 2:10, the transmission loss became more pronounced. This is because the higher refractive index of the hybrid nanofibers coating on fiber surface alters the effective RI distribution of the cladding modes, and the phase matching conditions is altered, particularly under high doping concentration in perovskite material. As a result, the total internal reflection condition at the cladding-surrounding medium interface is not satisfied, causing the guided modes in the cladding to transition into radiation modes.

**Figure 3:**
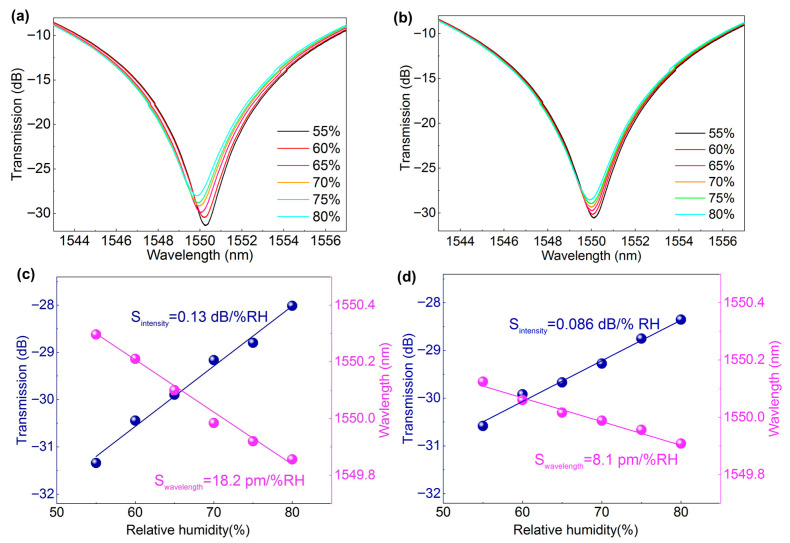
Spectral evolutions and the corresponding fitting curves of LPG1 modified with quasi-2D perovskite/PAN (1:10) hybrid nanofibers under electrospun time of 50 s (**a**,**c**) and 3 min (**b**,**d**), respectively.

We also prepared the humidity-sensitive films with varying thicknesses by adjusting the electrospun time. Figure 3a,b depicts the humidity-induced spectral evolutions of the LPG1 (doping ratio of 1:10) wrapped with quasi-2D perovskite/PAN hybrid fibers under electrospun times of 50 s (with film thickness of ~6.8 μm) and 3 min (with film thickness of ~11.3 μm), respectively. It was observed that both the wavelength and amplitude of the resonant dip near 1550 nm change as RH increasing from 50 to 80% tracking the resonant dip of the transmitted spectra of two gratings, the detailed RH sensitivities were plotted. As shown in the fitting curves in Figure 3c, the LPG1 with a thinner film exhibited a blue wavelength shift, and the sensitivity was 18.2 pm/%RH (R^2^ = 0.988), and the transmission loss sensitivity was 0.13 dB/%RH (R^2^ = 0.990). By comparison, the LPG1 with a thicker film shown in Figure 3d exhibited a smaller wavelength shift, and the sensitivity was 8.1 pm/%RH (R^2^ = 0.982), and the transmission loss sensitivity was also reduced to 0.086 dB/%RH (R^2^ = 0.989). The above results indicated that the LPG1 coated with a 6.8 μm hybrid nanofibers film had better sensitivity. This can be explained by the fact that as the wrapping thickness increases, the porosity of the reticular nanofibers film decreases, thus affected water molecule permeation and their interaction with the embedded quasi-2D perovskite materials.

**Figure 4:**
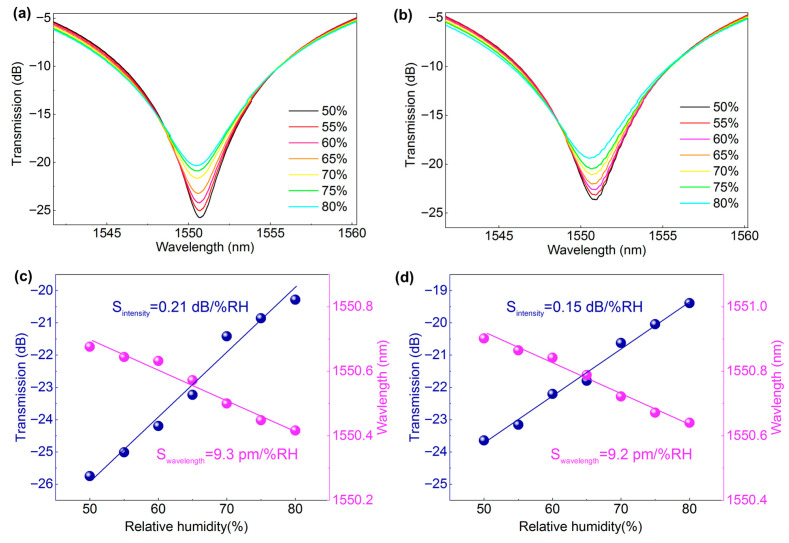
Spectral evolutions and the corresponding fitting curves of LPG2 modified quasi-2D perovskite/PAN (2:10) hybrid nanofibers under electrospun time of 50 s (**a**,**c**) and 3 min (**b**,**d**), respectively.

Figure 4a,b depicts the humidity-induced spectral evolutions of the LPG2 (doping ratio of 2:10) wrapped with quasi-2D perovskite/PAN hybrid fibers under electrospun times of 50 s and 3 min, respectively. As shown in Figure 4c, the resonant dip of LPG2 with a thinner film exhibited the wavelength sensitivity of 9.3 pm/%RH (R^2^ = 0.971), and the transmission loss sensitivity of 0.21 dB/%RH (R^2^ = 0.979). By comparison, the LPG2 with a thicker film exhibited the wavelength sensitivity of 9.2 pm/%RH (R^2^ = 0.984), and the transmission loss sensitivity of 0.15 dB/%RH (R^2^ = 0.991). The results from the two grating samples with different film thicknesses indicate that higher perovskite concentration and a thinner coating can enhance the sensor’s humidity sensitivity performance, with a best intensity sensitivity of 0.21 dB/%RH. It verified that as water molecules penetrating into the voids between the organic spacer layers of perovskites, this rapidly induces an expansion of the interlayer distance and the defect formation, and thus the alteration of the permittivity and complex RI of the film. Therefore, it finally leads to an abruptly decrease in grating coupling strength, meanwhile a blue shift in the transmitted dip of all the gratings.

**Figure 5:**
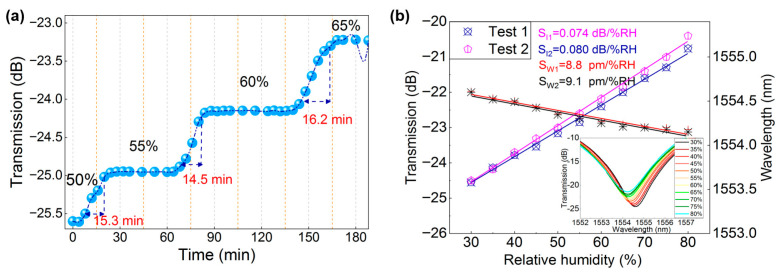
(**a**) Dynamic response characteristics and the stability of the humidity sensor; (**b**) Repeatability test of the LPG modified with quasi-2D perovskite/PAN (1:10) hybrid nanofibers in humidity range of 30–80%. Insert shows the spectral evolution of the sensor.

In order to check the dynamic response characteristics of the humidity sensor, we increased the relative humidity from 50% to 65% with an interval of 5%. Although both wavelength and intensity of the grating spectra respond to RH, the intensity is selected here due to the reason that wavelength of the grating dip is easily affected by surrounding environment. Therefore, the band loss variation in LPG2 (doping ratio of 2:10, film thickness of 6.8 µm) was recorded every 3 min, and the corresponding transmission intensity response are shown in Figure 5a. It can be seen that there is an average response time of ~15.3 min. Meanwhile, by maintaining the humidity level at 60% for 60 min, the sensor’s response stability was monitored. The curves demonstrated that there is almost no significant perturbation for the dip intensity, which confirms the good stability performance of the device. Finally, the two repeatability tests of the proposed sensor were performed in a wider RH range of 30–80%. Two grating samples with quasi-2D perovskite/PAN (1:10) hybrid nanofibers were fabricated and tested in the same condition. It is seen from Figure 5b that the intensity sensitivities for two tests were 0.080 dB/%RH and 0.074 dB/%RH, meanwhile the wavelength sensitivities were 8.8 pm/%RH and 9.1 pm/%RH, respectively. The results demonstrated good reproducibility of the sensor during fabrication and testing. However, due to the irreversible hydrolysis degradation of the perovskite materials in humid environments, the sensor’s performance cannot be recovered after exposure, limiting its use in repeatability measurement of humidity monitoring. Future work will focus on enhancing environmental stability through encapsulation strategies and material composition optimization, as well as expanding the applicability of the sensor in areas requiring high-performance humidity sensing devices. Table 1 shows the comparison data between various fiber humidity sensors. It indicates that our proposed LPG sensor shows good intensity sensitivity. Owing to the straightforward fabrication process and low cost of the moisture-sensitive materials, the proposed RH sensor is suitable for use as a disposable humidity indicator, especially in high-humidity environments.

## 4. Conclusions

In summary, this study developed an LPG humidity sensor based on a quasi-2D perovskite/PAN hybrid nanofibers film prepared by electrospinning technique. By monitoring the changes in the resonance dip of the long-period fiber grating, the sensor enables real-time detection of relative humidity, demonstrating an intensity sensitivity of 0.21 dB/%RH and a wavelength sensitivity of 18.2 pm/%RH. This approach provides a novel way to dynamically observe the structural and optical behavior of perovskite materials under humidity exposure, and also extends the application of optical fiber sensing technology to humidity monitoring.

## Figures and Tables

**Table 1:** Comparison of the sensing performance of different humidity sensors.

Structure	Sensing Material	Sensitivity	Response Time	Refs.
S-taper fiber	SiO_2_/PEG	78.7 lx/%RH	19 s	[31]
LPG	PDMS	7473 pm/%RH	Not specified	[32]
Multimode fiber-no core fiber (MMF-NCF)	Ag/MgF_2_/PDMS	0.5683 nm/%RH	Not specified	[33]
FBG	GQDs	3.26 pm/%RH	5.6 min	[34]
Hollow core fiber (HCF)	GO	0.12 dB/%RH	Not specified	[35]
LPG	Quasi-2D Perovskite/PAN	18.2 pm/%RH and 0.21 dB/%RH	~15.3 min	This work

## Data Availability

The original contributions presented in this study are included in the article. Further inquiries can be directed to the corresponding author.
