# Correction: Feng et al. Long Period Grating Modified with Quasi-2D Perovskite/PAN Hybrid Nanofibers for Relative Humidity Measurement. *Nanomaterials* 2026, *16*, 99

**DOI:** 10.3390/nano16030186

**Published:** 2026-01-30

**Authors:** Dingyi Feng, Changjiang Zhang, Syed Irshad Haider, Jing Tian, Jiandong Wu, Fu Liu, Biqiang Jiang

**Affiliations:** 1 MOE Key Laboratory of Material Physics and Chemistry under Extraordinary Conditions, State Key Laboratory of Porous Metal Materials, Shaanxi Basic Discipline (Liquid Physics) Research Center, and School of Physical Science and Technology, Northwestern Polytechnical University, Xi’an 710129, China; nupzcj@mail.nwpu.edu.cn (C.Z.); syed.irshad@mail.nwpu.edu.cn (S.I.H.); tianj@mail.nwpu.edu.cn (J.T.); fu.liu@nwpu.edu.cn (F.L.); bqjiang@nwpu.edu.cn (B.J.); 2 State Key Laboratory of Solidiffcation Processing, Center for Nano Energy Materials, School of Materials Science and Engineering, Northwestern Polytechnical University and Shaanxi Joint Laboratory of Graphene, Xi’an 710129, China; jiandong.wu@mail.nwpu.edu.cn

In the original publication [1], references 11, 12, and 31–39 were incorrectly attributed. References 36–39 were removed, and references 11, 12, and 31–35 have now been corrected as follows:

11. Deleau, C.; Seat, H.C.; Bernal, O.; Surre, F. High-sensitivity integrated SiN rib-waveguide long period grating refractometer. *Photonics Res*. **2022**, *10*, 564.
12. Saha, N.; Kumar, A. A novel dual resonance long period waveguide grating based highly sensitive refractive index sensor with reduced temperature sensitivity. *Opt. Commun*. **2020**, *474*, 126092.
31. Qi, Y.; Li, J.; Chen, Y.; Zhu, B.; Zhou, X.; Xiao, X.; Gu, Z.; Qian, J.; He, C.; Lai, M.; et al. Differential fiber optic humidity sensor based on superhydrophilic SiO_2_/polyethylene glycol composite film withlinear response. *Opt. Fiber Technol*. **2025**, *90*, 104150.
32. Wu, H.; Li, L.; Liu, H.; Ke, H.; Tu, H.; Huang, Y. Ultrasensitive fiber humidity sensor based on mode transition sensitized LPFG with low refractive index coating. *IEEE Sens. J.* **2025**, *25*, 39665–39672.
33. Song, M.; Jing, X.; Guo, P.; Yin, Z.; Li, S. SPR fiber optic sensor based on MMF-NCF doped with MgF_2_ for dual-parameter measurement of temperature and humidity. *Opt. Express* **2025**, *33*, 1869–1882.
34. Hou, J.; Dai, J.; Zhang, F.; Yang, M. Advanced Fiber-Optic Relative Humidity Sensor Based on Graphene Quantum Dots Doped Polyimide Coating. *IEEE Photon. Technol. Lett.* **2022**, *34*, 725–728.
35. Cheng, A.; Wang, C.; Xu, J.; Zhang, P.; Zheng, Y.; Dai, S. Humidity Sensor Based on a Hollow Core Fiber Anti-Resonant Reflection Optical Waveguide. *Photonic Sens*. **2025**, *15*, 250202.

With this correction, the Table 1 was corrected accordingly. The corrected Table 1 appears below.

The authors state that the scientific conclusions are unaffected. This correction was approved by the Academic Editor. The original publication has also been updated.

## Figures and Tables

**Table 1:** Comparison of the sensing performance of different humidity sensors.

Structure	Sensing Material	Sensitivity	Response Time	Refs.
S-taper fiber	SiO_2_/PEG	78.7 lx/%RH	19 s	[31]
LPG	PDMS	7473 pm/%RH	Not specified	[32]
Multimode fiber-no core fiber (MMF-NCF)	Ag/MgF_2_/PDMS	0.5683 nm/%RH	Not specified	[33]
FBG	GQDs	3.26 pm/%RH	5.6 min	[34]
Hollow core fiber (HCF)	GO	0.12 dB/%RH	Not specified	[35]
LPG	Quasi-2D Perovskite/PAN	18.2 pm/%RH and 0.21 dB/%RH	~15.3 min	This work

## References

[1] Feng D., Zhang C., Haider S.I., Tian J., Wu J., Liu F., Jiang B. (2026). Long Period Grating Modified with Quasi-2D Perovskite/PAN Hybrid Nanofibers for Relative Humidity Measurement. Nanomaterials.

